# A Novel Skull Clamp Positioning System and Technique for Posterior Cervical Surgery: Clinical Impact on Cervical Sagittal Alignment

**DOI:** 10.1097/MD.0000000000000695

**Published:** 2015-05-01

**Authors:** Nodoka Manabe, Takachika Shimizu, Tetsu Tanouchi, Keisuke Fueki, Masatake Ino, Naofumi Toda, Kanako Itoh, Kenji Shirakura

**Affiliations:** From the Department of Orthopaedic Surgery (NM, TS, TT, KF, MI, NT, KI), Gunma Spine Center (Harunaso Hospital), Kamitoyooka, Takasaki; and Department of Rehabilitation Medicine (KS), Gunma University Graduate School of Medicine, Showa-machi, Maebashi, Gunma, Japan.

## Abstract

A prospective radiographic study.

The purpose of this study was to analyze whether a novel skull clamp positioning system and technique is useful for obtaining good, quantitative cervical sagittal alignment during posterior cervical surgery.

Different surgical procedures depend on cervical spine positioning. However, maneuver of the device and cervical position depends on the skill of the operator.

This study included 21 male and 10 female patients with cervical spondylotic myelopathy and ossification of the posterior longitudinal ligament of the cervical spine, undergoing posterior cervical surgery using the novel skull clamp positioning system. The average patient age was 68.6 years (range: 56–87 years). The novel system has a scale to adjust the neck position and to enable intended cervical sagittal alignment. First, the patient was placed on the operating table in the prone position with preplanned head–neck sagittal alignment (neutral position in general). The head was rotated sagittally, and the head was positioned in the military tuck position with the novel device that was used to widen the interlaminar space. After completing the decompression procedure, the head was rotated again back to the initial preplanned position. During this position change, the scale equipped with the device was useful in determining accurate positions. The C0-C1, C0-C2, C1-C2, C2-C7, and C0-C7 angles were measured on lateral radiographs taken pre-, intra-, and postoperatively.

This novel system allowed us to obtain adequate, quantitative cervical sagittal alignment during posterior cervical surgery. There were no clinically significant differences observed between the pre- and postoperative angles for C1-C2 and C2-C7.

Sagittal neck position was quantitatively changed during posterior cervical surgery using a novel skull clamp positioning system, enabling adequate final cervical sagittal alignment identical to the preplanned neck position.

## INTRODUCTION

Cervical spine positioning technique is important during posterior cervical decompression and fusion. It is well known that a slightly flexed position of the cervical spine can widen the interlaminar or interspinous space, which is useful for a posterior cervical approach and decompression. However, corrective surgery and repair of the extensor musculature after cervical decompression need to bring the head–neck position back into the anatomical or preplanned position.^1^

Different surgical procedures depend on cervical spine positioning; therefore, it is difficult to conduct the entire posterior cervical surgery in the same cervical position. Various methods exist for positioning the cervical spine during posterior cervical surgery, including Mayfield head holders or tongs (Ohio Medical Instrument Co, Cincinnati, Ohio) with traction,^2^ 4 different attachments to the OSI Jackson table (Mizuho OSI, Union City, California),^2,3^ and positioning cushions.^4^ However, maneuver of the device and cervical position depends on the skill of the operator because it is difficult to hold a quantitatively good cervical position.

To the best of our knowledge, there has been no large study that has analyzed obtaining of a good, quantitative cervical sagittal alignment during posterior cervical surgery. Shimizu developed and reported cervical positioning for posterior cervical surgery using halo-vest fixation,^5^ and Fueki et al reported the development of a novel skull clamp positioning device, referred to as the GSS Head Grip Arc System (Johnson & Johnson KK, Japan).^6^ We have operated over 900 cases of different types of craniocervical, cervical, and cervicothoracic disorders using this novel device, from 2002 to 2014 at our hospital.

The purpose of the present study was to analyze whether the novel skull clamp positioning system and technique can be useful in obtaining good, quantitative cervical sagittal alignment during posterior cervical surgery.

## MATERIALS AND METHODS

### Characteristics of the Novel Skull Clamp Positioning Device

The novel skull clamp positioning system is a device for posterior surgery of craniocervical, cervical, and upper thoracic disorders. To achieve intended cervical sagittal alignment, this device has a scale to adjust the neck position.

**FIGURE 1:**
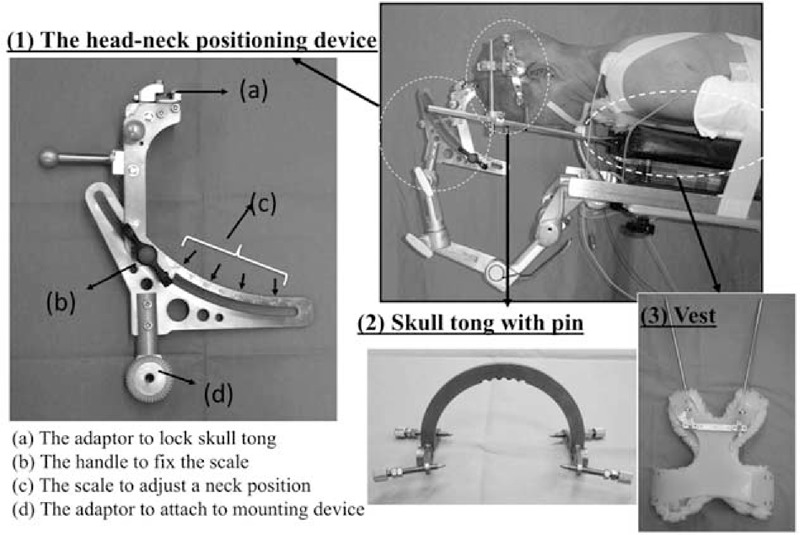
The novel skull clamp positioning device. The device comprises 3 main parts: (A) the head–neck positioning device to adjust neck position; (B) the skull tong to provide rigid cranial fixation; and (C) the vest to maintain head, neck, and body horizontally aligned. The scale reads from −15° to 35° in 5° increments. In the present study, the device scale was rotated sagittally from a 0° to 30° angle.

The device has 3 main parts: the head–neck positioning device; the skull tong to provide rigid cranial fixation; and the vest to maintain the head, neck, and body horizontally aligned. In addition, the head–neck positioning device has 4 main parts: an adaptor to lock the skull tong; a handle to fix the scale; a scale to adjust the neck position; and d) an adaptor to attach the mounting device to the operating table. The scale reads from −15° to 35° in 5° increments (Figure 1). In the present study, we rotated the device scale sagittally, from a 0° to 30° angle. However, we could select any angle of the scale at any point during the surgery.

### The Method for Using This Skull Clamp Positioning Device

**FIGURE 2:**
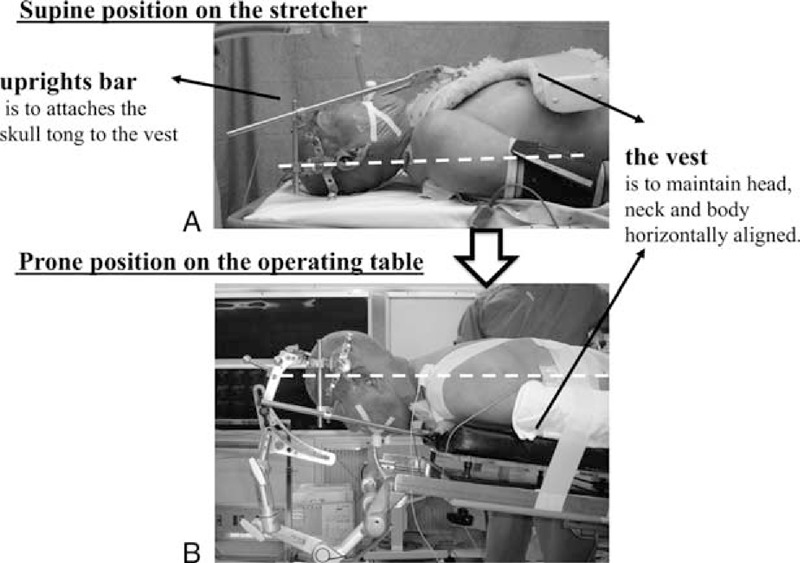
Patient positioning. First, the patient was positioned supine on the stretcher, the skull tong was fixed on the head using skull pins and the vest was mounted over the body in a preplanned head–neck position (A). The patient-tong-vest assembly was then turned 180° to be in the prone position and placed on the operating table (B).

First, the patient under general anesthesia was placed supine on the stretcher. The skull tong was then fixed onto the head using skull pins, and the vest was mounted over the body to enable the preplanned head–neck position (Figure 2A). The patient-tong-vest assembly was then turned 180° to be in the prone position and was placed on the operating table. The skull tong was then adapted to the head–neck positioning device (neutral position in general) (Figure 2B). Finally, the head was rotated sagittally, and the head was positioned in the military tuck position with the novel device that was used to widen the interlaminar space.^7,8^ To ensure safety and maintain the head, neck, and body horizontally aligned, the skull tong was attached to the anterior part of the vest via 2 upright bars (Figures 1 and 2).

### Patients

**FIGURE 3:**
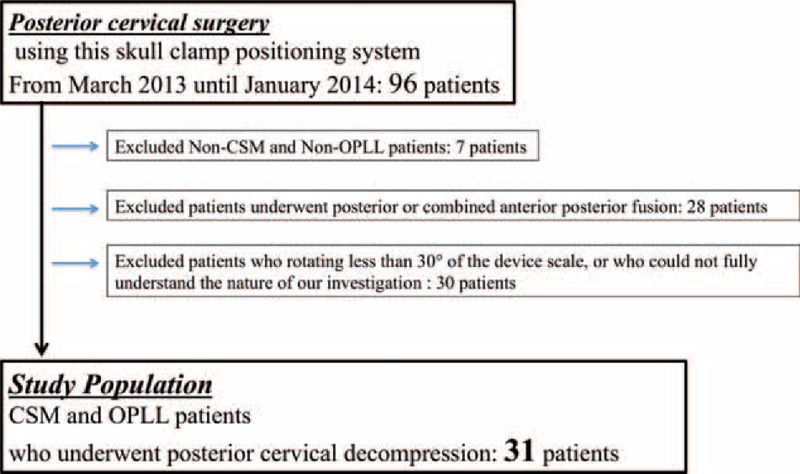
Flow chart for the selection of the study population. This study consisted of 31 patients with CSM (n = 27) and OPLL of the cervical spine (n = 4) undergoing posterior cervical decompression using the novel skull clamp positioning system. CSM = cervical spondylotic myelopathy; OPLL = ossification of the posterior longitudinal ligament.

Between March 2013 and January 2014, 96 patients underwent posterior cervical spine surgery using this skull clamp positioning system at our hospital. Of these 96 patients, 7 had atlantoaxial subluxation with malformed cervical vertebrae and a multiple-operated neck, 28 had undergone posterior cervical fusion or combined anterior posterior fusion, and 30 patients who had undergone posterior cervical surgery rotating less than 30° of the device scale, or who could not fully understand the nature of our investigation were excluded from the current analysis (Figure 3). Written informed consent to participate in the present study was obtained from all participants after sufficient explanation.

This study consisted of 31 patients with cervical spondylotic myelopathy (n = 27) and ossification of the posterior longitudinal ligament of the cervical spine (n = 4) undergoing posterior cervical decompression (modified en block laminoplasty) using the novel skull clamp positioning system. Twenty-one men and 10 women had an average age of 68.6 ± 10.8 years at the time of surgery. The average duration of the follow-up period was 1.3 ± 0.3 years (range: 1–1.8 years). All patients were positioned via the novel skull clamp positioning system described above. All patients underwent posterior cervical decompression according to the following levels: C2-T2 (n = 1), C3-C7 (n = 25), C3-T1 (n = 4), C4-T1 (n = 1) (Table 1). Neurologic evaluations were performed before and after the surgery according to the criteria that have been proposed by the Japanese Orthopaedic Association (JOA; 17 = maximum score).

**TABLE 1:**
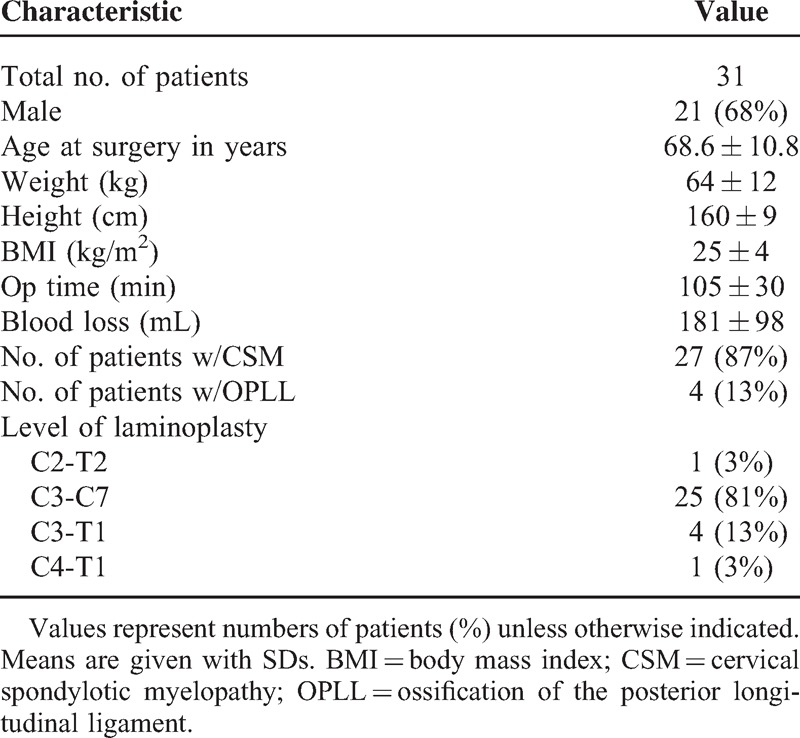
Demographic and Clinical Characteristics of 31 Patients Who Underwent Cervical Laminoplasty

### Alignment Using the Novel Device

**FIGURE 4:**
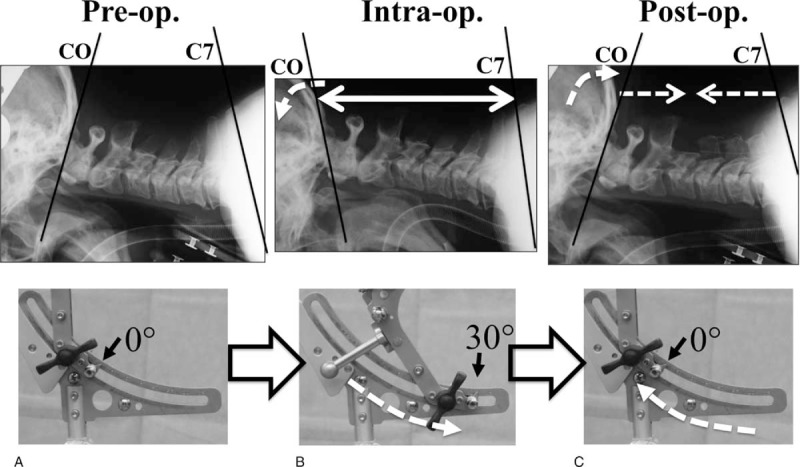
Novel device scale setting. (A) In the preoperative position, a preplanned head–neck position was ensured with the device scale at 0°. (B) In the intraoperative position, the military tuck position was ensured with the scale at 30°. (C) In the postoperative position, the initial preplanned position was ensured again with the scale reading 0°. Note that relative positional relationship between the head axis of rotation and the trunk was stable during this procedure.

First, the patient was placed on the operating table in the prone position with preplanned head–neck sagittal alignment and the device scale reading 0° at this point (preoperative position). Following which, the head was rotated sagittally and the head–neck position was changed to the military tuck position using the device to widen the interlaminar space with the scale reading 30° at this point (intraoperative position). After finishing the decompression procedure, the head was rotated again back to the initial preplanned position with the scale reading 0° at this point (postoperative position) (Figure 4).

C0-C1, C0-C2, C1-C2, C2-C7, and C0-C7 angles were measured on the lateral radiographs taken pre-, intra-, and postoperatively. The cervical range of motion, from pre- to intraoperative angle (dC0-C1, dC0-C2, dC1-C2, dC2-C7, and dC0-C7) and from pre- to postoperative angle (DC0-C1, DC0-C2, DC1-C2, DC2-C7, and DC0-C7), was calculated.

### Statistical Analyses

To identify differences between each time point, a paired sample *t* test was used. When the data were normally distributed, the Wilcoxon signed ranks test was used. In addition, 95% confidence intervals (CI) were calculated for each range of motion. All analyses were performed using SPSS 22.0.0 for Mac. All the reported *P* values were 2 tailed, with differences reported as significant if *P* < 0.05.

## RESULTS

The new system enabled us to obtain adequate, quantitative cervical sagittal alignment during posterior cervical surgery. The average JOA scores were 9.8 ± 2.4 points before the surgery, 12.2 ± 2.7 points a month after the surgery, and 14.0 ± 1.9 points at the final follow-up examination. The average recovery rate was 35.9% ± 9.6% one month after the surgery and 62.9 ± 16.3% at the final follow-up evaluation. No patients experienced any complications during the surgery.

**FIGURE 5:**
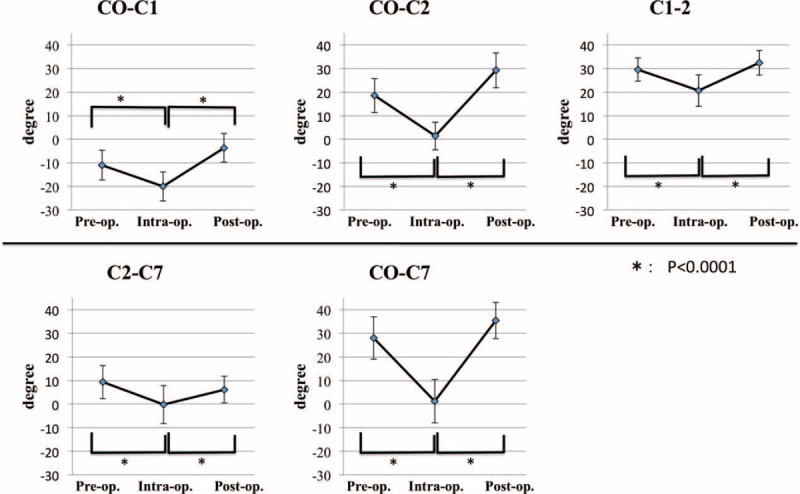
Pre-, intra-, and postoperative angles. The intraoperative angles of C0-C1, C0-C2, C1-C2, C2-C7, and C0-C7 led to a greater decrease compared with the preoperative angles. The decrease from pre- to intrao perative angle was statistically significant (*P* < 0.0001). The postoperative angles led to a greater increase compared with intraoperative angles. The increase from intra- to postoperative angle was statistically significant (*P* < 0.0001).

**FIGURE 6:**
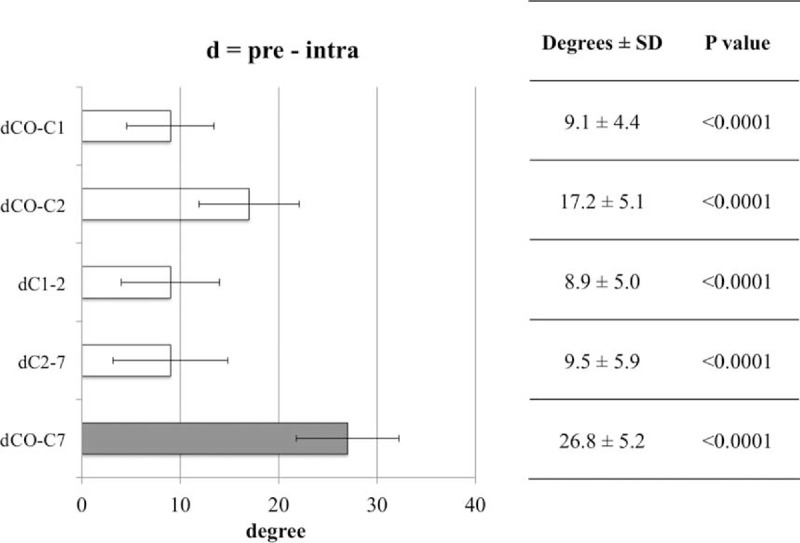
The cervical range of motion of the C0-C7 angle, from pre- to intraoperative position, was approximately 27°. The ratio of dC0-C2 to dC2-C7 was 2:1, and the ratio of dC0-C1:dC1-C2:dC2-C7 was 1:1:1, when the scale was rotated sagittally from 0° to 30°. SD = standard deviation.

The intraoperative angles for C0-C1, C0-C2, C1-C2, C2-C7, and C0-C7 led to a greater decrease compared with the preoperative angles, when the device scale was rotated from 0° to 30°. The decrease from pre- to intraoperative angle was statistically significant (*P* < 0.0001) (Table 2, Figures 5 and 6). These data showed that the interlaminar or interspinous space could be widened using the novel device.

**Table 2:**
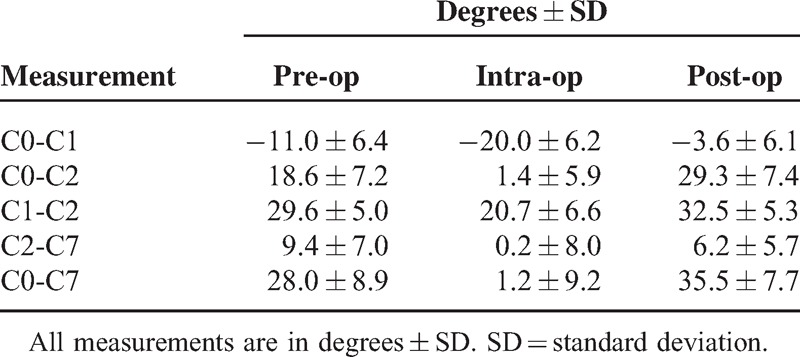
The Angles Measured on the Lateral Radiographs

The postoperative angles for C0-C1, C0-C2, C1-C2, C2-C7, and C0-C7 led to a greater increase compared with the intraoperative angles when the device scale was rotated from 30° to 0° again. The increase from intra- to postoperative angle was statistically significant (*P* < 0.0001) (Figure 5). These data show that the interlaminar or interspinous space could be narrowed.

We calculated the mean difference in degree and 95% CI of pre- and postoperative cervical sagittal alignment to assess reproducibility. We defined that if the mean difference and 95% CI were less than 5°, there was no significant clinical difference. In accordance with this, there was no significant clinical difference between DC1-D2 (difference 2.9°, 95% CI 1.6°– 4.2°) and DC2-D7 (difference −3.2°, 95% CI −4.9°–1.3°).

**FIGURE 7:**
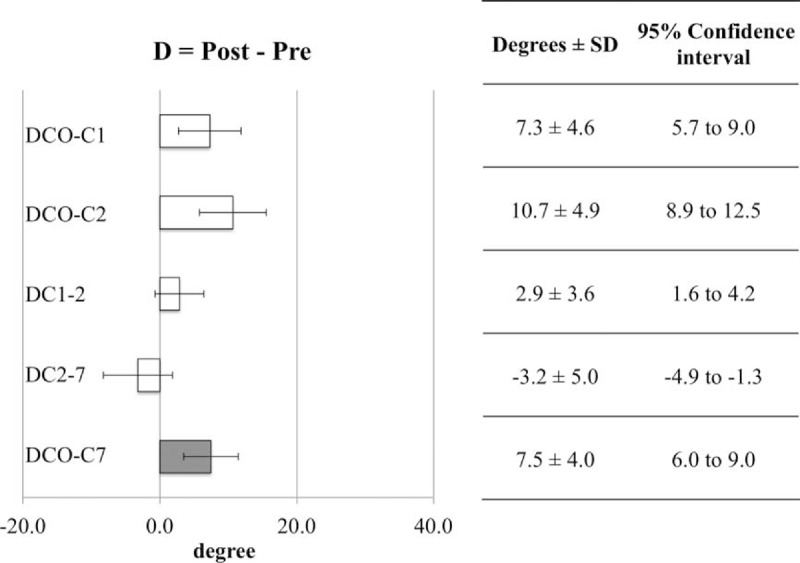
There was no significant clinical difference between DC1-C2 and DC2-C7. We defined that if the mean difference and 95% confidence interval were less than 5°, there was no significant clinical difference. SD = standard deviation.

Conversely, significant clinical differences were observed between DC0-C1 (difference 7.3°, 95% CI 5.7°– 9.0°), DC0-C2 (difference 10.7°, 95% CI 8.9°–12.5°), and DC0-C7 (difference 7.5°, 95% CI 6.0°–9.0°) (Figure 7).

### Case Study

**FIGURE 8:**
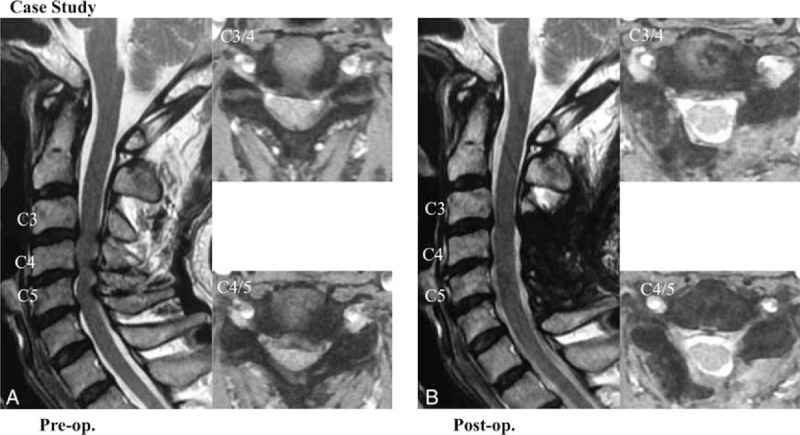
Preoperative (A) and postoperative (B) T2-weighted sagittal MR imaging in a 72-year-old man with multilevel cervical spondylotic myelopathy.

A 72-year-old man presented with progressive numbness of the extremities and tingling of both shoulders. A magnetic resonance (MR) image was obtained before the C3–C7 laminoplasty in this patient with multilevel cervical spondylotic myelopathy (A). An MR image that was obtained 3 months postoperatively shows adequate decompression of the cord (B). The JOA scores in this patient were 10.5 before surgery, 13.5 one month postoperatively, and 15 at the final follow-up examination. The recovery rates were 46.2% one month after the surgery and 69.2% at the final follow-up examination (Figure 8).

**FIGURE 9:**
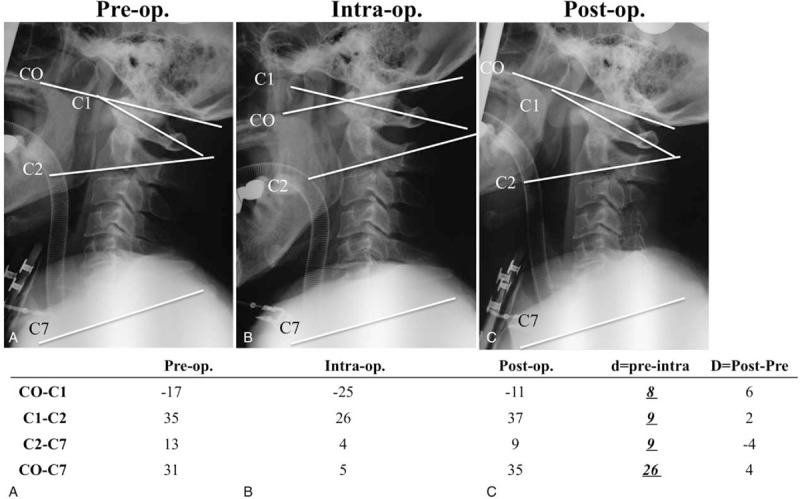
In this case, when the scale was rotated sagittally, from 0° to 30°, the cervical range of motion for the C0-C7 angle, from pre- to intraoperative position, was 26°, with a ratio for dC0-C1:dC1-C2:dC2-C7 of 1:1:1.

In this case, when the scale was rotated sagittally, from 0° to 30°, the cervical range of motion for the C0-C7 angle, from pre- to intraoperative position, was 26°, with a ratio for dC0-C1:dC1-C2:dC2-C7 of 1:1:1. In addition, we obtained quantitative C1-C2 and C2-C7 alignment between pre- and postoperative situations (Figure 9).

## DISCUSSION

### Changes in Cervical Sagittal Alignment From Pre- to Intraoperative Head and Neck Position

Hyperextension of the cervical spine can cause narrowing of the spinal canal due to buckling, invagination of the ligamentum flavum, and shingling of the laminae in patients with posterior abnormality.^4,9–11^ Furthermore, it is well known that cervical malalignment, after occipito-cervico-thoracic fusion, may cause dysphagia or dyspnea.^12–14^ Therefore, cervical spine positioning technique is important during posterior cervical decompression and fusion.

In the present study, when the scale was rotated sagittally, from 0° to 30°, each interlaminar or interspinous space was widened. The cervical range of motion for the C0-C7 angle, from pre- to intraoperative position, was approximately 27°, with a ratio for dC0-C2 to dC2-C7 of 2:1 and ratio for dC0-C1:dC1-C2:dC2-C7 of 1:1:1 (Figures 5 and 6). The new system allowed the occipital bone, which may be the biggest obstruction during posterior cervical surgery, to be kept away from the surgical field efficiently, enabling adequate, quantitative cervical sagittal alignment. Furthermore, the new system is useful for the surgery of occipital-atrantoaxial complex.

### Assessment of Repeatability From Pre- to Postoperative Sagittal Alignment of the Cervical Spine

We successfully obtained quantitative C1-C2 and C2-C7 alignment between pre- and postoperative situations; however, the postoperative C0-C1 and C0-C2 angles were increased significantly compared with the preoperative angles, resulting in an increase in the C0-C7 angle. Increased angles were considered to be influenced by an original greater cervical range of motion for C0-C1, C0-C2. Other reasons for the increase may include surgical invasion and stretching of the posterior cervical muscle.

These results corresponded with our previous experience, whereby when we performed occipito-cervical or occipito-thoracic fusion, we always took an x-ray to ensure enhanced cervical sagittal alignment. Accordingly, the intra-to-postoperative scale range of motion was 5° to 10°, which was smaller than the pre-to-intraoperative scale range of motion, to adjust for occipito-cervical alignment.

Using the novel device, we could select any angle of the scale at any stage of the surgery. In the present study, however, we rotated the device scale sagittally from 0° (preoperative) to 30° (intraoperative), and after completing the decompression procedure, from 30° (intraoperative) to 0° (postoperative). Therefore, if we want to adjust the postoperative C0-C1 or C0-C2 angle to the preoperative angle, we would need to rotate the intra-to-post scale range of motion 5° to 10° smaller than that of the pre-to-intra range of motion.

In conclusion, sagittal neck position was quantitatively changed during the posterior cervical surgery using the novel skull clamp positioning system, enabling adequate final cervical sagittal alignment identical to the preplanned neck position. C1-C2 and C2-C7 angles were obtained quantitatively by rotating an equivalent amount on the device scale. C0-C1 and C0-C2 angles may be obtained quantitatively by downregulating the device scale slightly. We would certainly consider using this technique during future cervical posterior surgery.
